# The Non-Linear Path from Gene Dysfunction to Genetic Disease: Lessons from the MICPCH Mouse Model

**DOI:** 10.3390/cells11071131

**Published:** 2022-03-28

**Authors:** Konark Mukherjee, Leslie E. W. LaConte, Sarika Srivastava

**Affiliations:** 1 Fralin Biomedical Research Institute at VTC, Roanoke, VA 24016, USA; lacon001@vtc.vt.edu (L.E.W.L.); sarika_srivastava@vtc.vt.edu (S.S.); 2 Department of Psychiatry, Virginia Tech Carilion School of Medicine, Roanoke, VA 24016, USA; 3 Department of Basic Science Education, Virginia Tech Carilion School of Medicine, Roanoke, VA 24016, USA; 4 Department of Internal Medicine, Virginia Tech Carilion School of Medicine, Roanoke, VA 24016, USA

**Keywords:** CASK, MICPCH, pontocerebellar hypoplasia, pathogenesis

## Abstract

Most human disease manifests as a result of tissue pathology, due to an underlying disease process (pathogenesis), rather than the acute loss of specific molecular function(s). Successful therapeutic strategies thus may either target the correction of a specific molecular function or halt the disease process. For the vast majority of brain diseases, clear etiologic and pathogenic mechanisms are still elusive, impeding the discovery or design of effective disease-modifying drugs. The development of valid animal models and their proper characterization is thus critical for uncovering the molecular basis of the underlying pathobiological processes of brain disorders. MICPCH (microcephaly and pontocerebellar hypoplasia) is a monogenic condition that results from variants of an X-linked gene, *CASK* (calcium/calmodulin-dependent serine protein kinase). *CASK* variants are associated with a wide range of clinical presentations, from lethality and epileptic encephalopathies to intellectual disabilities, microcephaly, and autistic traits. We have examined *CASK* loss-of-function mutations in model organisms to simultaneously understand the pathogenesis of MICPCH and the molecular function/s of CASK. Our studies point to a highly complex relationship between the potential molecular function/s of *CASK* and the phenotypes observed in model organisms and humans. Here we discuss the implications of our observations from the pathogenesis of MICPCH as a cautionary narrative against oversimplifying molecular interpretations of data obtained from genetically modified animal models of human diseases.

## 1. The Complexity of the Disease Process and Utility of Genetically Modified Animals

Key to the discovery and identification of disease-modifying agents is knowledge of the disease process. Disease onset and progression proceed through multiple steps, including etiology (initiation of the causal mechanism of the disease), pathogenesis (process by which the disease develops), structural changes in the affected organs, and finally, functional changes that have clinical significance [1]. Pathophysiology is far more chaotic and disordered than normal physiology due to the breakdown of homeostatic mechanisms. Because diagnoses are made only after the onset of functional changes (the last step described above), investigating the upstream disease process requires the generation of valid animal models for human disorders. Unfortunately, other than disorders caused by obvious extrinsic insults such as trauma, infections, and toxins, clear etiology has not been established for the majority of commonly occurring brain disorders.

Monogenic disorders with a defined etiology thus may provide a window to probe how molecular defects affect organ systems and lead to symptomatology. Even with monogenic conditions, however, interpretations are fraught with challenges; in many cases, disease manifestation is a consequence of indirect systemic changes and not the mere loss of molecular function, making it hard to recapitulate disease phenotypes in animal models like mice, which have biology distinct from humans. Heavily investigated monogenic conditions like β-hemoglobinopathies underscore this complexity, whereby the presenting symptoms vary not only with difference in variants but also with the same variant due to biological variability [2,3]. Differences in gene regulation also limit the utility of animal models, again as exemplified by the β-globin (*HBB*) gene [4]. 

Monogenic brain disorders produce complex behavioral phenotypes which are, in many cases, uniquely human. Overall, replicating brain disorders in animal models has been difficult. The biological differences inherent between species impact the external validity of animal models for brain disorders, making translation of laboratory findings rare and of minimal impact [5,6]. Furthermore, behavioral analysis in animal models of monogenic conditions can produce highly variable results [7,8]. Gene variants in the brain can produce complex pathophysiology which does not necessarily reveal the molecular function of the affected gene but instead represents systemic dysfunction. This notion has been simplified within the field of bioinformatics where gene ontology (GO) is defined through three different hierarchical parameters: molecular function, cellular component, and biological process [9]. Even this simplistic breakdown, however, is often not sufficient to derive the relationship between the genotype and the observed phenotype of many monogenic disorders because it disregards systemic effects. Phenotypes often arise from non-cell-autonomous mechanisms, and even acute, cell type-specific genetic modifications can produce non-cell-autonomous effects [10,11]. Furthermore, identical genetic manipulation can produce contrasting cellular and molecular phenotypes in different brain regions [8,12].

Additionally, the relationship between gene function and phenotype is further complicated by genetic pleiotropy (gene variants affecting multiple traits in an unexpected manner), along with penetrance and expressivity of gene variants [13,14]. These phenomena are modulated by genetic background as well as stochastic processes, including epigenetic changes [15]. The genotype–phenotype gap is difficult to fill and requires a more rigorous understanding of organ systems and their interactions; in the central nervous system, such an understanding is far from complete [16,17]. 

Background effects and incomplete penetrance are also often observed in animal models. Additionally, there are confounds in animal models that originate from the methodology of producing genetic modifications [18,19]. Experimental designs with animal models are not often focused on addressing important questions such as the mechanism of pleiotropy, penetrance, or the role of genetic background, which might provide valuable insight into pathogenesis of genetic disorders. Instead, researchers are incentivized to suppress any heterogeneity of phenotype to derive a simplistic “molecular mechanism”. A consequence of this approach is that genetically modified animals are frequently phenotyped by using specific assays that are based on expectations of molecular function/s. The absence of a phenotype within these assays is neither interpreted nor pursued, whereas the presence of the expected phenotype reinforces the idea of a precise molecular mechanism. The role of systemic compensation and developmental plasticity is often overlooked [20]. Evidence, however, suggests that even specific defects like changes in synaptic physiology in animal models with genetic modification are likely to be the product of developmental plasticity [21]. Understanding the relationship of a gene’s molecular function to the phenotypes observed with specific gene variants may thus require unbiased investigations that employ multiple techniques on an appropriate animal model. Here we discuss our experience from investigation of a monogenic brain disorder. 

## 2. MICPCH and *CASK*-Linked Pathologies

MICPCH (microcephaly and pontocerebellar hypoplasia), a monogenic pediatric brain disorder, is associated with variants of *CASK* (calcium/calmodulin-dependent serine protein kinase). We have investigated the function/s of the CASK protein for nearly 15 years. Our investigation of MICPCH by using the *CASK* mutant mouse has required a broad lens to integrate our findings at both the molecular and organismal levels. We seek to simultaneously understand: (1) the molecular and cellular function of the CASK protein, and (2) how *CASK* loss produces MICPCH; we suspect that these two questions are not as related as is commonly assumed.

The human *CASK* gene was first described as a candidate locus for X-linked optic atrophy in 1998 [22]. Almost a decade later, Froyen et al. described a female with microcephaly and intellectual disability with a large deletion which also affected the *CASK* gene [23]. Although they proposed that *CASK* was the possible X-linked intellectual disability (XLID) gene, a clear association could not be arrived at, because other genes were included in the deletion. Soon after, it was confirmed that *CASK* indeed was an XLID gene [24], and the complete description of *CASK*-linked pathology, which included disproportionate hindbrain affliction with cerebellar and brain stem hypoplasia, was described in five individuals [25]. Large-scale sequencing efforts also indicated that *CASK* indeed was an XLID gene and missense variants within the *CASK* gene were frequent among boys with XLID [26,27]. These boys typically present with variable XLID, with or without nystagmus. The boys are mostly normocephalic, and some of them inherit the variant from their mother [27]. Authors universally invoked a neurodevelopmental role for the purported CASK-Tbr1 interaction in neuronal migration as the mechanism for the new neurodevelopmental disorder designated MICPCH [25,28]. There was, however, little evidence of defective neuronal migration in other contexts of *CASK*-linked disorders that were not MICPCH but were often attributable to missense variants in *CASK*, such as normocephalic boys with XLID and even in FG syndrome [27,29]. Not all *CASK* missense variants induced destabilization of protein structure, suggesting that these variants likely affected specific CASK functions [30]. 

Loss-of-function *CASK* variants are more commonly seen in girls, likely due to early lethality in boys. Such loss-of-function variants manifest with MICPCH, global developmental delay, growth retardation, optic nerve hypoplasia/optic atrophy, and seizures in 40% of cases [31,32,33]. Although the disorder has been considered developmental, the observed microcephaly has been consistently described as postnatal and progressive [31,32]. A longitudinal study of clinical and radiological course indicates that at birth, the height and weight of the girls are typically normal and ~50% of individuals also have a normal occipitofrontal circumference (normocephalic). Affected girls develop head control normally, and motor delay is usually first detected between 3 and 6 months after birth [34]. Early in infancy, these girls do, however, display progressive microcephaly, growth retardation, and pontocerebellar hypoplasia (PCH) in magnetic resonance imaging (MRI). Most clinical indications thus suggest that embryonic and early fetal brain development in the setting of a *CASK* variant is not severely affected, and disease onset appears only in later stages of brain development, including the third trimester and postnatal period. 

In contrast to girls, who have two alleles of the *CASK* gene, hemizygous boys with *CASK* loss-of-function variants present with a more severe phenotype. In addition to MICPCH, they are also diagnosed with epileptic encephalopathies such as Ohtahara syndrome and West syndrome (infantile spasms) due to the presence of early-onset epilepsy and diagnostic EEG (electroencephalogram) patterns such as burst-suppression and hypsarrhythmia [35,36,37,38]. Boys with mosaic variants in *CASK* that arise during embryogenesis present with MICPCH, just like girls [33,35]. Overall the phenotype indicates that the role of the *CASK* gene itself does not have any sex-specificity, but rather that differences in phenotype between girls and boys arise simply due to the fact that in boys there is only one X-chromosome and hence one *CASK* gene. Analysis of boys with *CASK*-null variants suggests that the lethality observed in boys is due to thinning of the brain stem which leads to aberrant respiratory and deglutition reflexes manifesting systemically as central neurogenic respiratory failure, airway infection, and pneumonia [35,36,37]. A previously published histopathology of a posthumous *CASK*-variant case suggested that there could be defects in cortical and cerebellar layering, indicating a defect in neuronal migration. However, the decedent harbored a synonymous variant at the 305th position (K305K), indicating it may not have been a loss-of-function variant. The variant likely leads to defective splicing in a small fraction of mRNA (~20%), as demonstrated in an in vitro experiment [25]. Without direct molecular data from the decedent, it is not possible to rule out a potential missed dual diagnosis or a gain of function due to an aberrantly spliced transcript. In contrast, our autopsy study on a boy with a *CASK*-null variant (R27*) with a nonsense mutation at the beginning of the protein documented that, along with several other organs, the brain is small (~60% by weight) but exhibited normal gyrification, indicating appropriate migration of neurons. No specific defects were found in the vasculature or meninges. The hindbrain, including the cerebellum and brain stem, was found to be extremely hypoplastic, and histologically, a decrease in granule cells in the cerebellum and gliosis was noted; no hallmarks of defective neuronal migration were evident [37].

*CASK* variants can also be associated with nonspecific phenotypes in organs other than the brain [33,35,37]. In some cases, *CASK* variants have been associated with microcephaly without cerebellar hypoplasia [39]. It has proven difficult to perform definitive genotype–phenotype correlation studies of *CASK* variants, although it has been suggested that variants in the C-terminus are more likely to be correlated with nystagmus due to CASK’s interaction with FRMD7 [27,40]. A critical analysis of the relationship between nystagmus and specific *CASK* variants, however, indicates that nystagmus is incompletely penetrant and may be cerebellar in origin. Nystagmus can be associated with *CASK* variants in all regions of the protein [27,41,42]. Overall, *CASK* variants are associated with a wide spectrum of phenotypes, most consistently and notably affecting the nervous system. Within the brain, the hindbrain is disproportionately affected.

## 3. Interpretation of Findings from *CASK* Null Animals

The phenotypes of three commonly used animal models—worms, flies, and mice—without *CASK* are vastly different. Whereas *CASK*-null worms exhibit vulval defects with no neuronal phenotype [43,44] and *CASK*-null flies exhibit a change in locomotor behavior with no observable change in development or survival [45,46], *Cask*-null mice die within hours of birth [47] (Table 1). This underscores the necessity to interpret gene function data from animal models within the appropriate evolutionary biological niche. The vulval defect seen in *CASK*-null worms has been attributed to the molecular role of CASK in the Let-23 epidermal growth factor pathway [44]. In flies, the observed locomotor defect is not the result of specific defects in neuromuscular junctions, motor neurons, or premotor neuropil [46]. Overall, data from invertebrate animal models failed to uncover a role for CASK in any essential neuronal function such as neuron development or synaptic transmission.

**Figure 1:**
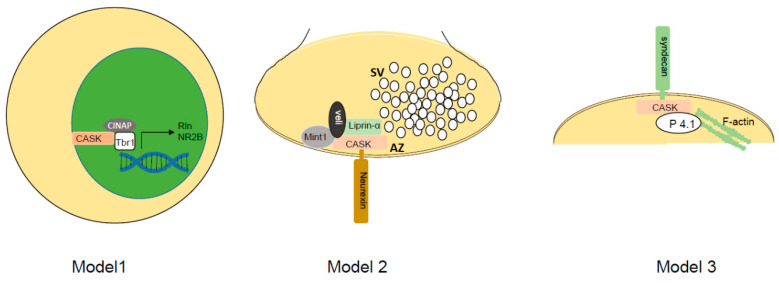
Working models of CASK molecular function. Model 1 shows CASK forming a complex with CASK-interacting nucleosome assembly protein (CINAP) and T-Box brain transcription factor 1 (Tbr1) to upregulate transcription of reelin and NR2b. Model 2 depicts CASK interacting with presynaptic adhesion molecule neurexin to nucleate a complex with Mint-1, veli, and the active zone (AZ) organizer liprin-α. SV = synaptic vesicle cluster. Model 3 depicts CASK interacting with transmembrane proteoglycan called syndecan on the postsynaptic side. Interaction of CASK with protein 4.1 (P4.1) via its hook motif allows nucleation of actin and spine maintenance.

In mice, deletion of *Cask* produced cleft palate with 80% penetrance in both sexes [47], which has not been routinely observed in humans. *Cask*-null mice were investigated within the confines of the existing assumptions about CASK’s biochemical function/s (Figure 1). CASK is a multidomain protein belonging to the MAGUK (membrane-associated guanylate kinase) family. From the N- to the C-terminus, CASK is composed of a CaM kinase (calcium/calmodulin-dependent protein kinase)-like domain, two L27 (lin-2, lin-7) domains, a PDZ (PSD95, dlg, ZO-1) motif, an SH3 (src homology 3) domain, and a GuK (guanylate kinase) domain [51]. Nestled in the hinge region between the SH3 and GuK domain is a hook motif [52]. Through its PDZ motif, CASK can interact with the presynaptic adhesion molecule neurexin [51] and with the post-synaptic transmembrane protein syndecan [53,54] (Figure 1); CASK has thus been presumed to function in both pre- and post-synaptic compartments. Indeed CASK, through its N-terminus, can interact with a variety of molecules including Mint1 [55], Caskin [56], and the active zone organizer liprin-α [57], further suggesting that CASK could be a presynaptic scaffolding molecule. Through its C-terminal GuK domain, CASK interacts with the T-box protein Tbr1, and it has been suggested that CASK may function within the nucleus by promoting expression of the extracellular molecule reelin, which is critical for neuronal migration, and NR2b, which is a subunit of postsynaptic NMDA receptors (Figure 1) [58]. Finally, some data suggest that CASK plays a role in cell proliferation and cell polarization [59,60,61]. 

Surprisingly, the brain from the *Cask*-null neonatal mouse is indistinguishable from the wild type, with proper lamination [47]. The loss of CASK in mice thus does not significantly alter the neurodevelopmental trajectory; on the contrary, increased apoptosis in the thalamus after birth was observed. Synapse formation and synaptic ultrastructure also remained unchanged in the absence of CASK, although some synaptic impairment is observed [47] (discussed below). None of these results, however, explain the mechanism of MICPCH or even the observed lethality. It is crucial to note here that genetic ablation of the CASK-Tbr1 interaction does not generate any of the phenotypic characteristics of MICPCH [62]. Deletion of *CASK* in mammals has thus far failed to uncover a role for CASK in core neuronal functions such as passive and active membrane properties or action potential-dependent neurotransmission [47]. Intriguingly, the thalamus of *Cask*-null neonatal mice exhibits a higher rate of neuronal loss in the form of apoptosis [47].

At a fundamental level, the findings from invertebrate and mammalian models are not inconsistent. Just as observed in humans, the lethality in the *Cask*-null mouse can be attributed to central neurogenic respiratory failure [47]. The central rhythmic pattern of the respiratory cycle is evolutionarily recent [63] and thus unlikely to affect survival among invertebrates. Although consistent at a fundamental level, the observations from *CASK*-null animal models suggest that organismal and behavioral phenotypes with similar genetic variants are likely to be highly divergent within the animal kingdom.

## 4. Further Revelations from *CASK*-Mutant Mice

Although it is hard to overcome the barrier of external validity due to biological differences generated through speciation, several approaches can improve the quality of information gathered from experiments in animal models. Most important is molecular conservation of the gene being perturbed. The CASK protein is highly conserved in the animal kingdom, with the murine and human CASK proteins being almost identical (~99% identity). Within the gene, the intron–exon boundaries are also conserved, although there is an expansion in the intronic length of the vertebrate gene [64]. The high degree of conservation of CASK indicates that it may have a highly conserved molecular function between mouse and human. We therefore set out to uncover the mechanism responsible for MICPCH by using a murine model.

We first examined if it was at all possible to replicate the MICPCH phenotype by using a murine model. We designed and produced *Cask* heterozygous knockout mice. Due to the X-linkage, this study was confined to female animals. The *Cask*^+/−^ mice recapitulated many of the features of MICPCH, including postnatal microcephaly, disproportionate cerebellar hypoplasia, optic nerve hypoplasia, scoliosis, and even occasional seizures [49,65,66]. Despite some defects in locomotor coordination and mild changes in visual contrast sensitivity, these mice thrived, are fertile, and take care of their pups adequately. They display motor and spatial learning [49,67]. In short, despite gross similarities in the brain structural phenotype between human MICPCH subjects and the murine model, the behavioral phenotypes are strikingly milder in mice. We examined the electrical activity of the *Cask*^+/−^ mouse cortex and retina by using EEG and ERG (electroretinogram) and found it to be indistinguishable from wild-type littermates [66,67].

An immediate consequence of *Cask* being an X-linked gene is that *Cask*^+/−^ mice are mosaic for CASK expression due to random X inactivation. This feature of the *Cask* gene makes it easier to ask questions about cell-autonomous effects without sophisticated genetic manipulation. Any bias towards the selection of cells that express CASK would lead to a secondary skewing of X-chromosome inactivation. Surprisingly, our experiments revealed that the *Cask*^+/−^ brain expresses ~50% of CASK compared to wild type, indicating the absence of secondary skewing and the presence of an equal number of CASK(+) and CASK(−) cells. These results suggest that any decrease in neuronal numbers in MICPCH mostly arise due to a non-cell-autonomous effect, which is surprising, given that CASK is an intracellular protein. Our findings were confirmed with a more sophisticated technique of single-cell qRT-PCR, which found more than 50% of neurons in the *Cask*^+/−^ brain lack CASK. Loss of CASK thus does not increase neuronal loss in a cell-autonomous fashion [48]. 

We confirmed this observation by using three different sets of experiments in structures that are specifically affected by CASK loss. First, we deleted *Cask* from Purkinje cells in the developing cerebellum specifically. This manipulation did not affect either the survival of Purkinje cells or locomotor function of the mice during the period of observation [49]. We next deleted *Cask* from more than 90% of retinal ganglion cells (RGC) whose axons form the optic nerve. This manipulation again did not affect RGC maturation or survival [67]. Finally, to test whether deletion of CASK had any effect on maturation and migration of granule cells of the cerebellum, we deleted *Cask* from a subset of granule cell precursors within the rhombic lip. Again, we did not observe any change in the trajectory of development or migration of the granule cells that lacked CASK [49]. These data were consistent with our finding that although *Cask*^+/−^ mice display a reduced number of neurons in the inner granule layer, the external granular layer (where granule cells mature) remains unperturbed during the developmental phase [49]. These findings led us to make the following inferences: (1) the effect that CASK loss has in MICPCH is non-cell-autonomous (in other words, survival or dysfunction of an individual neuron is not directly tied to its own expression levels of CASK but rather to collective expression levels of CASK in the surrounding tissue), and (2) CASK loss does not affect neuronal development. Indeed both the brain and optic nerve in mice show a size reduction compared to littermates around one week after they are born [49,65]. Careful observation of retinal ganglion cells indicates that the number of neurons is not reduced until much later than the first week of postnatal life [65]. 

With no evidence for a defect in neuronal development, we next decided to determine if CASK loss results in the degeneration of formed neurons beginning in the third trimester. In the human brain without CASK, we observed astrogliosis as well as microgliosis, both hallmarks of neuronal damage and neuronal loss. In the setting of a human brain without CASK, the presumptive neuronal loss occurs during the early development of the cerebellum, making it hard to further interpret the results. To better address the question of degeneration in the setting of early development, we next synchronized the timing of *Cask* deletion with the completion of neuronal migration in the cerebella of mice. We observed that this manipulation leads to rapid atrophy of the cerebellum, specifically of the granule cell layer, beginning at 2 months [37]. The mice remain otherwise healthy but lose their balance completely. Interestingly, the loss of balance does not occur until cerebellar atrophy is present, indicating that the observed ataxia results from damage to the tissue rather than loss of CASK molecular function. Our careful study of *Cask*-mutant mice overall thus suggests that loss of CASK induces pathology in the form of neuronal injury and neuronal death and that behavioral phenotypes are most likely secondary to structural changes, similar to the general process described for natural diseases in our introductory section. 

## 5. The Molecular Function of CASK

CASK can interact with a large number of proteins, leading to the idea that CASK is a scaffolding molecule [52,55,56,68,69,70,71,72,73,74,75,76,77]. Mammalian CASK was identified due to its ability to bind neurexin [51]. CASK can also interact with other membrane proteins such as syndecan and syncam [53,54,77]. CASK forms an evolutionarily conserved tripartite complex with two other molecules—Mint1 (lin-3) and veli (lin-7) [55,78]. Although at first glance, these data support the notion that CASK might participate in the let-23 pathway, within neurons, this conserved complex is thought to be presynaptic. The interaction of Mint-1 with CASK is competitive with other molecules such as Caskin, Tiam, and liprin-α [79,80,81]. The interaction of CASK with liprin-α is specific to vertebrates. Liprin-α can also additionally interact with the CASK-neurexin complex, an interaction which is modifiable by phosphorylation of neurexins [79,81]. Due to specific substitutions in the primary structure, both the CaM kinase domain and GuK domain of CASK were initially assumed to be enzymatically inactive [82,83]. Careful analysis, however, indicated that the CASK CaM kinase domain was an unusual kinase inhibited by divalent ions, including the kinase co-factor magnesium [84,85]. Due to its slow activity, CASK can only phosphorylate its interacting partners, such as neurexin. Such phosphorylation events are likely to alter protein–protein interactions, as has been demonstrated for the CASK-neurexin-liprin-α interaction [79]. Although the CASK–neurexin interaction is typically described to be PDZ domain-mediated, biochemical experiments have indicated that the entire C-terminus region may be involved in this interaction [51]. The structural basis for this has been also described, demonstrating that the PDZ, SH3, and GuK domain form an integrated supradomain, which is required for the interaction between CASK and neurexin [86]. By analyzing *CASK* missense variants associated with MICPCH, we uncovered that disruption of this integrated C-terminus-mediated interaction, rather than the CASK-Tbr1 interaction, is more likely to be critical for inducing MICPCH [42]. This finding has been reproduced by another group [87]. However, variants throughout *CASK* are associated with developmental delay and MICPCH, demonstrating the difficulty of genotype–phenotype correlations at a protein structure level [30,41,42,88,89]. We have shown that many of the missense variants may actually lead to destabilization of the CASK structure and thus affect its overall function. 

Much of the available literature on CASK points to a putative synaptic function (Figure 1). As mentioned above, CASK can interact with the presynaptic protein neurexin and link it with active zone organizer liprin-α in a regulated manner. Further, liprin-α may regulate CASK turnover within the presynaptic active zone [90]. These function/s of CASK could be crucial for neurotransmission. Human-induced neurons with *CASK* variants showed a reduction in presynaptic protein levels, but this data is difficult to interpret because the research team found the opposite effect when knocking down *CASK* in wild-type neurons. Further complicating the effort to look at effects on gene expression was the fact that the proportion of the neuron type (excitatory vs. inhibitory) that developed was altered in induced cells with *CASK* variants [91]. These data exemplify the difficulties of interpreting data acquired from cultured neurons in general and induced neurons specifically. On the postsynaptic side, CASK may interact with syndecan or CamKII via its interaction with Tbr1, and it can also alter the function of NMDA receptors by upregulating the NR2b subunit [53,58,92]. CASK thus may function in spinogenesis [93], synaptic plasticity, and neurotransmission. Indeed Mori et al. demonstrated that in the absence of CASK in post-synaptic neurons, there is a reduction in action potential-independent inhibitory current frequency and an increase in action potential-independent excitatory current frequency [48]. These data are consistent with findings in neurons from constitutive *Cask* knockout mice [47]. In the *Cask*^+/−^ mouse, we have demonstrated a decrease in release sites in the retinogeniculate connections [65]. We have also uncovered a decrease in the level of all active zone proteins and a concomitant increase in many glutamatergic post-synaptic proteins, further strengthening the notion of a synaptic role for CASK [66]. Additionally, CASK can interact with CNTNAP2 to stabilize dendritic arbors, specifically in cortical GABAergic inhibitory neurons [94]. 

Could all these putative functions of CASK underlie the MICPCH phenotype? The cerebellar hypoplasia in MICPCH most predominantly affects glutamatergic granule cells rather than GABAergic Purkinje cells [49,50]. Furthermore, the CNTNAP2 knockout mice do not display lethality or cerebellar hypoplasia [95]. Granule cells degenerate before formation of the inner granule layer and parallel fiber connections to Purkinje cells. In fact almost all synapses on Purkinje cells in MICPCH were found to be perisomatic and likely belonging to climbing fibers [50]. Further, abolishing neuronal activity or synaptic transmission in granule cells does not produce cerebellar degeneration ([96,97] and personal communication with Michael Fox). Finally, our experiment with acute deletion of CASK did not produce locomotor incoordination, indicating that the synaptic function(s) of CASK may not directly translate into symptomatology [50]. Thus although CASK may participate in many synaptic functions which could contribute to some phenotypes seen with *CASK* variants, there are likely to be additional cellular functions of CASK that are responsible for the phenotypes seen in MICPCH.

A fraction of CASK expressed by a cell is cytosolic and exists as part of larger protein complexes [49]. To fully describe the protein complexes associated with CASK, we have performed extensive immunoprecipitation and pulldown experiments. First, we performed such experiments in the fly model and were able to confirm many of the previously known CASK interactions, including with Mint, veli, neurexin, and CaMKII [98]. Surprisingly, we found that CASK likely interacts with different proteins in different neurons, allowing for functional diversification of CASK. A common set of proteins that immunoprecipitated with CASK were mitochondrial [98]. Strikingly, none of the mitochondrial genome-encoded proteins were precipitated with CASK, indicating that CASK was not interacting with mature complexes present in mitochondria, but rather with mitochondrial proteins outside of the mature mitochondria. We also performed pulldown experiments from the rat brain and found that CASK was part of four distinct complexes: (1) synaptic, (2) ribosomal/protein chaperone, (3) cytoskeletal, and (4) mitochondrial [66]. CASK protein–protein interactions may thus be conserved in different animal models.

We next tested whether these interactions have any physiological relevance. We examined transcriptomic changes in the *Cask*^+/−^ mouse brain by using an RNAseq approach; only ~100 transcripts exhibited significant changes, of which many were extracellular matrix (ECM) molecules. Changes in the ECM are noted in other neurodegenerative conditions [99] and may reflect ECM turnover. And although CASK purportedly interacts with Tbr1, strikingly, we did not observe changes in the transcript levels of the proteins proposed to be regulated by the CASK-Tbr1 complex (reelin and Nr2b). Others have also observed that Tbr1 may not regulate reelin expression in the cerebellum [100].

**Figure 2:**
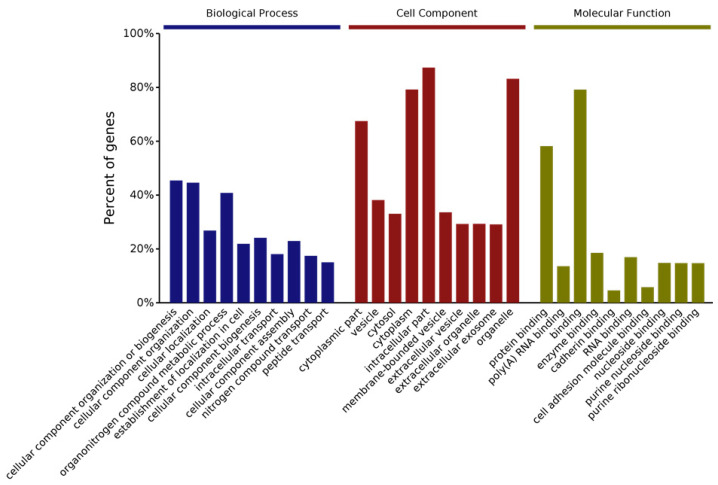
Gene ontology (GO) analysis of protein changes from whole brain of *Cask*^+/−^ mice compared to wild-type littermates. iTRAQ quantitative proteomic analysis was performed to evaluate global changes at protein level. Bar graphs shows top 10 GO annotation categories including biological process (blue), cell component (red), and molecular function (green).

**Figure 3:**
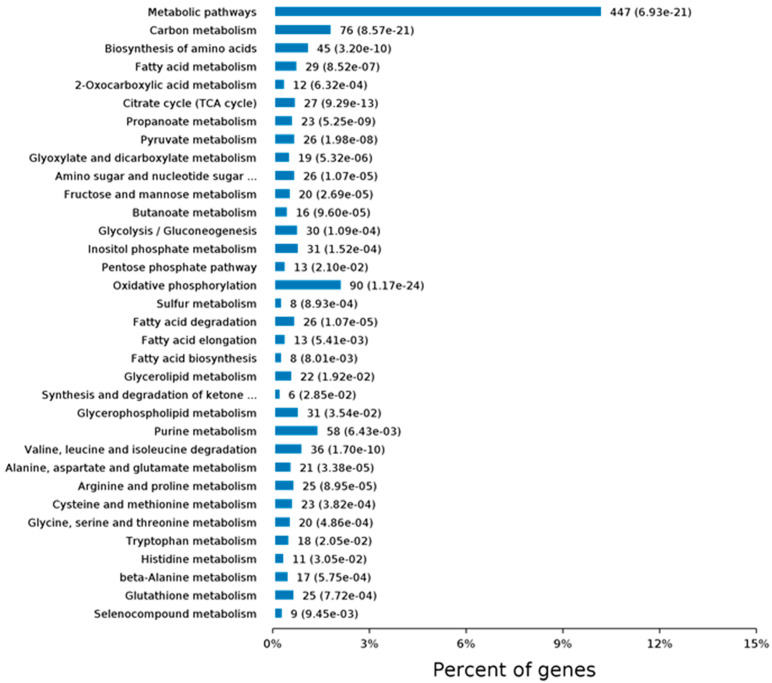

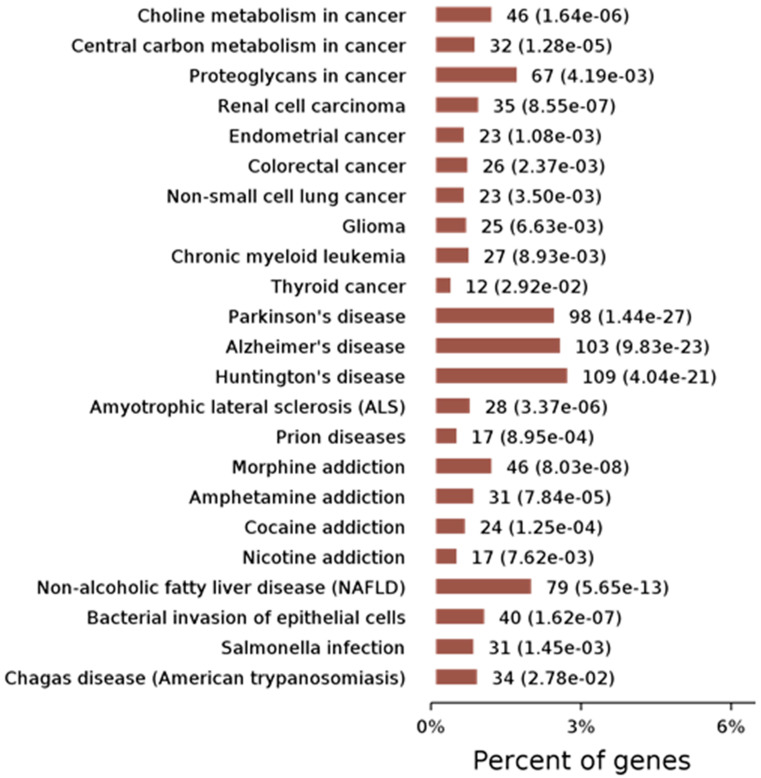
KEGG (Kyoto Encyclopedia of Genes and Genomes) pathway analysis of protein changes from whole brain of *Cask*^+/−^ mice compared to wild-type littermates. iTRAQ quantitative proteomic analysis was performed to evaluate global changes at protein level. KEGG pathway analysis was performed on the molecules. Significantly affected pathways are shown in metabolism (upper panel) and human disease pathway (lower panel) are shown. The full figure can be found in the supplemental file.

We have also examined changes in protein levels by using quantitative proteomics. More than 500 proteins are differentially expressed in *Cask*^+/−^ mice. Interestingly, these differentially expressed proteins fall into the same categories occupied by the proteins with which CASK forms complexes [66]. Ninety-nine of the proteins belong to the mitochondrial group, affirming our earlier findings of a link between CASK and the mitochondrion and suggesting that CASK regulates mitochondrial function in addition to synaptic function [66]. Gene ontology or GO analysis, which broadly classifies proteins based on their properties (molecular function, cellular component, or biological process), was used to look at what types of proteins change with CASK loss. In the “molecular functions” category, most affected proteins are related to protein binding (including enzymes) and nucleic acid binding. CASK loss affects proteins associated with a wide range of cellular components, both membranous and cytoplasmic, consistent with CASK’s distribution. And finally, according to the GO classification, the biological processes most affected with CASK loss range from metabolism to cellular organization and intracellular transport of proteins (Figure 2). Kyoto Encyclopedia of Genetics and Genomics (KEGG) pathway analysis of protein changes with CASK loss indicates that the highest significance is associated with neurodegenerative conditions such as Parkinson’s disease and the metabolic pathway of oxidative phosphorylation (Figure 3). These bioinformatics findings are corroborated by our experimental work, in which we indeed observed a loss of the substantia nigra pars compacta in the brain lacking CASK and also have biochemical evidence that mitochondrial respiration and glucose oxidation are reduced in the brains of *Cask*^+/−^ mice. In addition to metabolism, other fundamental pathways including protein translation and intra- and inter-cellular signaling are also affected by CASK loss (Figure 3).

The first CASK ortholog (C. elegans) to be discovered (lin-2) in 1978 was found to be a part of an evolutionarily conserved tripartite complex (with lin-7 and lin-10) [55,78], which may be localized to the Golgi complex [43,78,101]. In flies, CASK has been isolated with mitochondrial membranes [98]. Within the soma of a mammalian neuron, CASK can be found in a distinct punctate (organelle) distribution [54]. It has been suggested that CASK is localized to the endoplasmic reticulum, whereas its interactor Mint-1 (lin-10) has been localized to the Golgi complex and veli (lin-7) has been found on mitochondria [70,102,103]. Multiple studies have identified CASK as a probable constituent of mitochondria-associated membrane [104,105]. It is thus conceivable that CASK is involved in a more general protein folding and trafficking process, thereby affecting a wide range of functions including synaptic neurotransmission and metabolism [66]. This notion is in line with the ubiquitous expression of CASK in nearly all tissue types [51,106].

Several important questions remain to be answered about the molecular, cellular, and biological functions of CASK. For instance, does CASK have different functions in different cellular types? Experiments from Drosophila suggest that CASK may interact with different sets of proteins in different neuronal populations. The studies described here in-depth have all been done in brain tissue; CASK interactions may be even more diverse in different organs [98]. The cellular function of CASK is thus likely to be context-specific in different cell types, and the cumulative loss of these CASK functions contribute to the overall phenotype of MICPCH. The role of CASK throughout the body has been postulated to be impressively broad, including possible contributions to insulin secretion and signaling, cell proliferation, kidney development, heart conductivity, epithelial cell polarization, cancer biology, and sperm development, just to name a few [59,61,75,107,108,109,110], suggesting that CASK dysfunction leads to systemic effects that are hard to disentangle from each other. 

## 6. Reconciling CASK’s Molecular Function with Its Loss-of-Function Pathology

It is often not easy to correlate the pathology of a genetic disorder with the specific molecular function/s of the gene. For example, mitochondrial encephalopathies can have phenotypes that overlap substantially with those caused by variants in proteins that glycosylate macromolecules [111]. We are, however, able to provide a rough outline of the connection between genotype and phenotype from our studies of CASK. Loss of CASK produces PCH [31]. In most instances, PCH has been classified as a degenerative disorder with a prenatal onset [112]. At present, more than 11 subtypes of PCH have been described based on clinical and radiographic features. Many of the genes that have been tied to PCH are involved in tRNA function [112]. Other genes associated with PCH include the mitochondrial arginylyl tRNA synthetase (RARS2) and the exosome genes Exosc 3 and 8 [112]. Careful consideration of these associations strongly suggests that dysfunction in protein translation, mitochondrial metabolism, and exosomal dysfunction positively correlate with the occurrence of PCH. What remains to be uncovered is why defects in these particular functions specifically affect the hindbrain. 

PCH with CASK loss also begins prenatally in the third trimester. As seen with PCH, proteomic changes in the brains of mice with heterozygous deletion of *Cask* feature mitochondrial and exosomal proteins, as well as proteins involved in translation (Figure 2 and Figure 3 and supplemental figure). It is possible that loss of CASK destabilizes these vital cellular functions and thus produces PCH. PCH caused by CASK variants has traditionally been considered non-degenerative. Some authors have gone further and concluded that CASK-linked PCH arises via a unique mechanism because there are no observed deficits in supratentorial structures (it is important to note, however, that many of those conclusions were drawn from the investigation of female heterozygous cases) [113,114]. Unlike other PCH-associated genes, *CASK* is an X-linked gene subject to random inactivation, meaning that in females with a heterozygous *CASK* variant, at least 50% of their brain cells have a normal complement of CASK (mosaic status); in this setting, the disorder produced by *CASK* variant is non-progressive in nature. We have demonstrated, however, that total deletion of *Cask* produces progressive cerebellar atrophy and that supratentorial atrophy is, in fact, present in *CASK*-linked PCH in males [35,36]. Much evidence thus strongly suggests that *CASK*-linked PCH is mechanistically similar to other forms of PCH. In line with this notion, there is substantial overlap in the clinical and neuroradiological presentations of CASK and TSEN54-related disorders [115]. Moreover, *CASK* variants have been associated with PCH 2, 3, and 4 [113,116]. *CASK* variants may, in fact, be one of the most common causes of PCH [115]. Clearly, more human case studies are necessary to gather definitive evidence of neurodegeneration in these disorders.

A confounding property of CASK’s pathogenesis is that neuronal loss due to CASK loss-of-function occurs in a non-cell-autonomous fashion. How the dysfunction produced within cells by loss of CASK gives rise to non-cell-autonomous neuronal loss remains unclear. It is not known whether PCH associated with other genes also occurs due to similar non-cell-autonomous effects. It is pertinent to mention that non-cell-autonomous toxicity has been proposed to underlie many neurodegenerative conditions [117]. Further research on CASK is likely to shed light on the mechanism of non-cell-autonomous toxicity and the hindbrain’s vulnerability to CASK dysfunction. In addition to PCH, *CASK* variants also cause microcephaly. Microcephaly is a co-morbidity of several other PCH types and not unique to CASK variants [118]. 

## 7. Remaining Questions

Many questions remain to be answered regarding *CASK*-linked disorder and CASK’s molecular function. Although related, two of the most pressing questions need to be approached as independent questions: (1) what are the major molecular/cellular function/s of CASK? and, (2) what is the mechanism of non-cell-autonomous toxicity in CASK deficiency? In contrast to other MAGUKs like PSD-95, a significant amount of CASK is not membrane-bound but rather is soluble. Furthermore, CASK is bestowed with an unusual protein kinase activity. The molecular function of CASK is thus likely to be highly complex and dynamic. It is critical to tackle the important biochemical tasks of identifying additional substrates for CASK-kinase activity and then describing the impacts of such phosphorylation.

From the perspective of pathology, the most important question to be pursued is the mechanism of non-cell-autonomous neuronal loss associated with *CASK* variants. Likely related to this question is why the hindbrain is disproportionately afflicted (although we also observe that mid-brain structures like the substantia nigra pars compacta and retinal cells can be affected by CASK loss). In some ways, that *CASK*-linked pathology is degenerative in nature provides a positive outlook. Because microcephaly in *CASK*-linked pathology progresses postnatally, there may be a temporal window when therapeutic intervention might prevent or slow further brain cell loss. Regression, even in adolescence, has also been observed in some cases of MICPCH [119], again offering the tantalizing possibility that a therapeutic approach might prevent such decline under conditions when degeneration is known to progress. The potential benefits of intervention might extend even further given that non-cell-autonomous toxicity could also affect functioning of the remaining neurons; reduction of such toxicity, especially when coupled with high-intensity rehabilitative measures [120], might offer real hope for a positive impact on functional outcomes. 

## 8. Conclusions

The ability to modify a gene in a controlled fashion in animal models is a powerful technology both for facilitating biomedical discoveries as well as for understanding fundamental biology. Interpretations from such experiments, however, are unlikely to be amenable to linear models in the form of the ”gain and loss of function” that we often seek and are satisfyingly comprehensible. Although deletion of CASK failed to affect core neuronal functions in both invertebrate and vertebrate animal models, the associated phenotypes in each case demanded additional unbiased scrutiny. Our research has been driven by the molecular, cellular, and phenotypic consequences of *Cask* gene deficiency and how those consequences relate to MICPCH; we have not restricted our research focus to the role that CASK plays in specific neuronal functions such as synapse formation, neurotransmission, or short- and long-term plasticity. This approach has already provided valuable clues regarding the pathogenesis of MICPCH and its relationship to other neurodegenerative conditions. Furthermore, our approach has clarified that CASK deficiency adheres to the fundamentals of the pathological basis of disease, meaning that functional loss only manifests after the disease process has produced structural changes [1]. Our work raises an uncomfortable yet essential question regarding the study of genetically modified animal models using techniques such as imaging or electrophysiology: are we studying the molecular function of the gene involved, or are we instead examining the downstream effects of a pathological process?

## Figures and Tables

**Table 1:** *CASK* variant phenotypes observed in animal models, with references.

Animal Model	Major Findings	Citation
C. elegans	Vulvaless phenotype. No other developmental or neuronal defect.	[43,44]
D. melanogaster	Reduced locomotion. No change in neuromuscular junction.	[45,46]
Constitutive *Cask* knockout mice	Death within hours of birth; cleft palate; normal-sized, well-laminated brains; increased neuronal death in thalamus. Increased excitatory synaptic miniature current frequency; decreased inhibitory synaptic miniature current frequency.	[47]
Constitutive heterozygous *Cask* knockout mice	Postnatal microencephaly; cerebellar hypoplasia; optic nerve hypoplasia; locomotor incoordination; scoliosis; occasional seizures. Increased excitatory synaptic miniature current frequency; decreased inhibitory synaptic miniature current frequency only in *CASK*-null cells.	[48,49]
*Cask* deletion in subset of cerebellar granule cells	No obvious phenotype.	[49]
*Cask* deletion in Purkinje cells	No obvious phenotype.	[49]
*Cask* deletion in all cerebellar granule cells	Cerebellar atrophy.	[50]

## Supplementary Materials

The following supporting information can be downloaded at: https://www.mdpi.com/article/10.3390/cells11071131/s1, Figure S1: KEGG (Kyoto encyclopedia of genes and genomes) pathway analysis of protein changes from whole brain of *CASK*^+/−^ mice compared to wildtype littermates.
