# Supplementary material for: The Non-Linear Path from Gene Dysfunction to Genetic Disease: Lessons from the MICPCH Mouse Model
Source: Cells. 2022 Mar 28;11(7):1131. doi: 10.3390/cells11071131 (PMC8997851; doi:10.3390/cells11071131)
Supplement: Supplementary file 1 [file cells-11-01131-s001.zip › cells-1593431-supplementary.pdf]

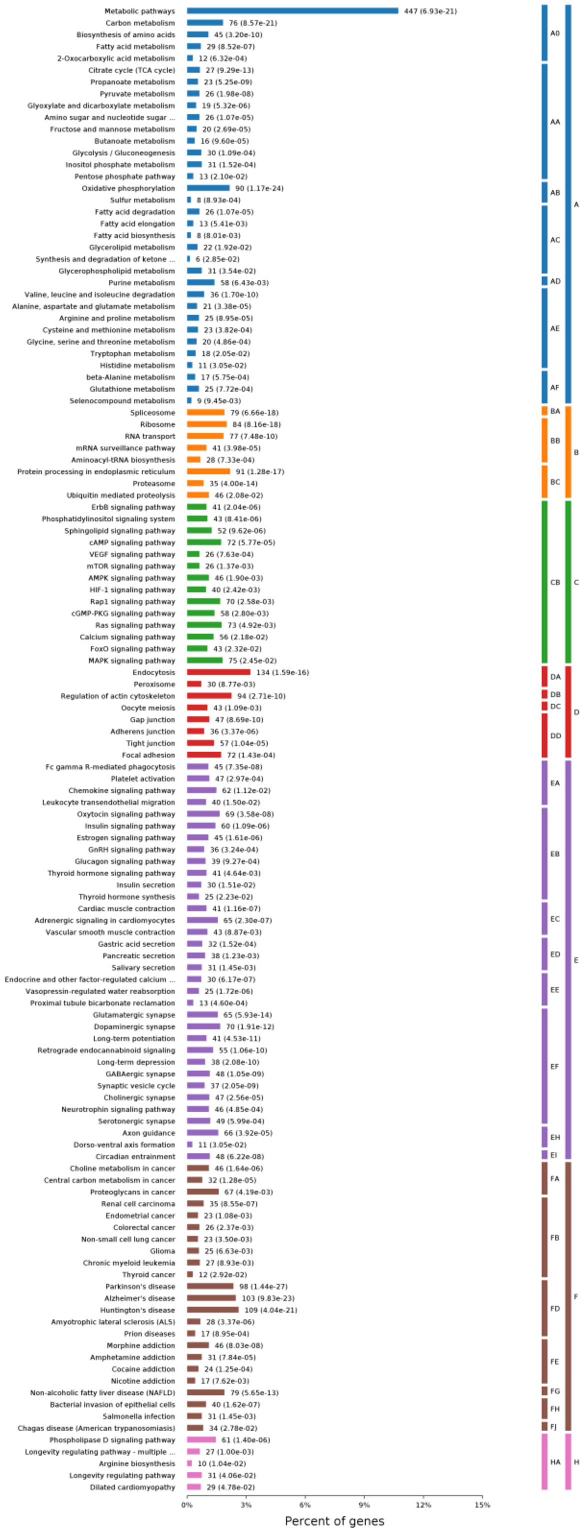

A : Metabolism
A0 : Global and overview maps
AA : Carbohydrate metabolism
AB : Energy metabolism
AC : Lipid metabolism
AD : Nucleotide metabolism
AE : Amino acid metabolism
AF : Metabolism of other amino acids

B : Genetic Information Processing
BA : Transcription
BB : Translation
BC : Folding, sorting and degradation

C : Environmental Information Processing
CB : Signal transduction

D : Cellular Processes
DA : Transport and catabolism
DB : Cell motility
DC : Cell growth and death
DD : Cellular community

E : Organismal Systems
EA : Immune system
EB : Endocrine system
EC : Circulatory system
ED : Digestive system
EE : Excretory system
EF : Nervous system
EH : Development
EI : Environmental adaptation

F : Human Diseases
FA : Cancers: Overview
FB : Cancers: Specific types
FD : Neurodegenerative diseases
FE : Substance dependence
FG : Endocrine and metabolic diseases
FH : Infectious diseases: Bacterial
FJ : Infectious diseases: Parasitic

H : Other and unknow
HA : Other and unknow

Figure S1. KEGG (Kyoto encyclopedia of genes and genomes) pathway analysis of protein changes from whole brain of *CASK*$^{+/-}$ mice compared to wildtype littermates. iTRAQ quantitative proteomic analysis was performed to evaluate global changes at the protein level. KEGG pathway analysis was performed on the proteins that exhibited differences between *CASK*$^{+/-}$ and wildtype mice. Significantly affected pathways are shown. The key for representation with alphabetical code is also provided.
